# NF‐kB signaling in cardiomyocytes is inhibited by sevoflurane and promoted by propofol

**DOI:** 10.1002/2211-5463.12783

**Published:** 2020-01-15

**Authors:** Keiko Oda‐Kawashima, Anna S. Sedukhina, Naoki Okamoto, Mariya lytvyn, Kimino Minagawa, Teppei Iwata, Toshio Kumai, Eri Sato, Eiichi Inada, Ayako Yamaura, Miki Sakamoto, Marta Roche‐Molina, Juan A. Bernal, Ko Sato

**Affiliations:** ^1^ Department of Pharmacogenomics St. Marianna University Graduate School of Medicine Kawasaki Japan; ^2^ Anesthesiology Division Graduate School of Medicine Juntendo University Bunkyo‐ku Japan; ^3^ Centro Nacional de Investigaciones Cardiovasculares (CNIC) Madrid Spain; ^4^ Department of Anesthesiology St. Marianna University School of Medicine Kawasaki Japan

**Keywords:** anesthesia, bioinformatics, myocardial remodeling, NF‐kB, propofol, sevoflurane

## Abstract

Both inhalational and intravenous anesthetics affect myocardial remodeling, but the precise effect of each anesthetic on molecular signaling in myocardial remodeling is unknown. Here, we performed *in silico* analysis to investigate signaling alterations in cardiomyocytes induced by inhalational [sevoflurane (Sevo)] and intravenous [propofol (Prop)] anesthetics. Bioinformatics analysis revealed that nuclear factor‐kappa B (NF‐kB) signaling was inhibited by Sevo and promoted by Prop. Moreover, nuclear accumulation of p65 and transcription of NF‐kB‐regulated genes were suppressed in Sevo‐administered mice, suggesting that Sevo inhibits the NF‐kB signaling pathway. Our data demonstrate that NF‐kB signaling is inhibited by Sevo and promoted by Prop. As NF‐kB signaling plays an important role in myocardial remodeling, our results suggest that anesthetics may affect myocardial remodeling through NF‐kB.

## Graphical abstract

The precise nature of the molecular alterations occurring after inhalation or intravenous anesthesia remains unclear. Here, *in silico* gene expression analysis of patient cardiomyocytes and a mouse animal model revealed that nuclear factor‐kappa B signaling is upregulated by sevoflurane and downregulated by propofol. Interestingly, this effect was observed only in the postanesthesia acute phase, not in the late phase.

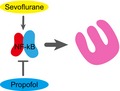

**Abbreviations**
ACM: arrhythmogenic cardiomyopathy
CABG: coronary artery bypass graft
DEG: differentially expressed gene
FDR: false discovery rate
LV: left ventricular
MM: M‐mode
NF‐kB: nuclear factor‐kappa B

Myocardial remodeling is a response of cardiomyocytes to stresses such as infarction, pressure overload, and cardiomyopathy. Importantly, myocardial remodeling affects heart function, which can eventually cause heart failure 1. Alterations in gene expression have been extensively studied to elucidate the mechanism underlying myocardial remodeling 2, 3. Gene clusters involved in myocardial remodeling, such as inflammation, fibrosis, and cardiomyocyte death, were identified in a series of studies 1, 2, 3. Nuclear factor‐kappa B (NF‐kB) signaling functions in myocardial remodeling by regulating inflammation and cell death 1, 4. Interestingly, short‐term activation of NF‐kB has a cardioprotective effect against hypoxia and reperfusion injury, while sustained activation promotes heart failure due to excessive inflammation 5, 6.

The clinical effects of different types of anesthetics on the heart have been well studied. Several meta‐analyses revealed that inhalational anesthetics, such as sevoflurane (Sevo), reduce morbidity and mortality after cardiac surgery 7, 8. The intravenous anesthetic propofol (Prop) elicits cardioprotective effects and decreases myocardial infarct size, troponin release, and mortality after cardiac surgery, ischemia, and reperfusion 9, 10, 11, 12. Thus, both inhalational and intravenous anesthetics exert cardioprotective effects. The optimal anesthetic for cardiac surgery has also been studied 8, 13, 14, 15. A meta‐analysis revealed that Sevo elicits beneficial effects on cardiac troponin I release, but not on cardiac function, length of intensive care unit stay, or duration of mechanical ventilation after cardiac surgery 8. The cardioprotective effects of anesthesia during surgery and reperfusion after ischemia have been investigated; however, signaling alterations related to myocardial remodeling in response to different types of anesthesia have not been studied.

A study investigated gene expression alterations in cardiomyocytes in response to inhalational and intravenous anesthesia using RNA sequencing 16. Comparison of the expression signature of all genes revealed that most signaling pathways are altered similarly in response to these two types of anesthetics, while three pathways are differentially regulated. However, as mentioned by the authors, this was an observational study and did not provide a direct link to a physiological event 16.

Here, we identified differentially expressed genes (DEGs) following the administration of inhalational or intravenous anesthesia and explored signaling alterations based on these DEGs. This analysis revealed that Sevo inhibits and Prop promotes NF‐kB signaling, which plays an important role in myocardial remodeling. These findings were confirmed in a mouse model.

## Materials and methods

### Bioinformatics analysis

Gene expression data [RMA‐normalized, natural scale values and 54 613 probes in http://www.ncbi.nlm.nih.gov/geo/query/acc.cgi?acc=GSE4386 available in the (HG‐U133_Plus_2) Affymetrix Human Genome U133 Plus 2.0 Array] were downloaded from ArrayExpress (E‐GEOD‐4386) 16. Gene expression was compared between the Sevo‐ and Prop‐administered groups as described previously 17. Briefly, natural values were converted to the Log_2_ scale. Data in the Sevo‐ and Prop‐administered groups were divided into two based on before or after OFF‐PUMP coronary artery bypass graft (CABG) surgery. Gene expression was compared between the two groups to identify DEGs using Significance Analysis of Microarrays Citation in R (two‐class unpaired method for the unpaired test and two‐class paired method for the paired test) 18. A DEG was defined as a gene with a false discovery rate (FDR) of < 0.05 and a LogFC of > 1 or < −1. The DEGs identified underwent core analysis in Ingenuity Pathway Analysis. A canonical pathway was defined as significantly altered when the *P*‐value was < 0.05.

### Animal experiments

All mice were housed at room temperature on a 12‐h light–dark cycle for at least 1 week prior to experimentation, and food and water were given *ad libitum*. Anesthesia was induced and maintained based on previous studies and titration experiments 19, 20. Specifically, anesthesia was induced with 5% Sevo and maintained with 3% Sevo for 3 h. For anesthesia using Prop, a working stock of 25 mg·mL^−1^ 2,6‐diisopropylphenol (Sigma‐Aldrich, St. Louis, MO, USA) diluted in intralipid (20% emulsion available at Sigma‐Aldrich) was prepared. Mice were intraperitoneally administered 600 mg·kg^−1^ Prop. Animals under anesthesia were kept on a heating element at 37 °C. The effect of anesthesia was confirmed by examining the pedal reflex every 5 min. Mice were kept under anesthesia using each type of anesthetic for ~ 3 h. For the analysis, mice were euthanized by CO_2_ inhalation.

### Statistical analysis

Gene expression was compared between Sevo‐ and Prop‐administered mice using the Mann–Whitney *U*‐test. Differences were considered significant when the two‐tailed *P*‐value was < 0.05.

### Study approval

The animal experiments were approved by the Animal Ethics Committee of St. Marianna University (approval number: 1712002).

### Immunohistochemistry and measurement of protein expression

Immediately after recovery from anesthesia, mice were euthanized and their hearts were dissected. Hearts were fixed in 10% formalin neutral buffer solution for more than 3 days. Paraffinized tissue sections were placed onto coated slides (6 µm) and deparaffinized using routine techniques 21. Endogenous peroxidase activity was blocked by treatment with phosphate‐buffered saline containing 3% H_2_O_2_ for 10 min. Sections were incubated with an anti‐p65 antibody for 60 min and then with an appropriate secondary antibody for 30 min to detect p65. The level of nuclear p65 was measured using imagej. (National Institutes of Health, Bethesda, MD, USA and the Laboratory for Optical and Computational Instrumentation, University of Wisconsin, Madison, WI, USA).

### Antibodies

The following antibodies were used at the specified dilutions: anti‐p65 antibody (D14E12; Cell Signaling Technology, Danvers, MA, USA; 1 : 400) and Alexa Fluor‐conjugated secondary antibodies (Thermo Fisher Scientific, Waltham, MA USA; 1 : 1000).

### Real‐time RT‐PCR

Quantitative real‐time RT‐PCR was performed using a StepOnePlus™ Real‐Time PCR System (Applied Biosystems, Warrington, UK). Total RNA was extracted using a RNeasy Mini Kit (Qiagen Sciences, Valencia, CA, USA), and cDNA was synthesized using PrimeScript™ RT Master Mix (Takara, Tokyo, Japan) according to the manufacturers’ protocols. Real‐time PCR was performed using Power SYBR Green PCR Master Mix (Applied Biosystems) and the following conditions: 10 min at 95 °C and then 40 cycles of 15 s at 95 °C and 60 s at 60 °C. Fold changes in expression were determined using the cycle threshold method. The relative abundance of specific genes was normalized against that of *β‐actin*. The following primer sequences were used: *β‐actin*, forward CATCCGTAAAGACCTCTATGCCA and reverse ATGGAGCCACCGATCCACA; *BMP4*, forward TAGTCCCAAGCATCACCC and reverse TCTCAGCGGCATCCAC; *CARD10*, forward GCGAGGTCTACCCCATTGTC and reverse CAACAGGCCCCTGATCTCAC; *TRAF5*, forward CCGACACCGAGTACCAGTTTG and reverse CGGCACCGAGTTCAATTCTC; *CXCL1*, forward ATGATCCCAGCCACCCGCTC and reverse TTACTTGGGGACACCTTTTAGC; *TNFR1*, forward ATCTGCTGCACCAAGTGCC and reverse TGCATGGCAGTTACACACG; *BMPR1B*, forward TCAATGTCGTGACACTCCCATTCCT and reverse TGCTGTACCGAGGTCGGGCT; *IL18*, forward GCCTCAAACCTTCCAAATCA and reverse TGGATCCATTTCCTCAAAGG; and *TLR2*, forward GCTGGATTTATCCAGGTGTG and reverse TCTCCACAGCCACCAGATTCT.

### Echocardiography acquisition

An expert operator blinded to the effects of anesthesia performed transthoracic echocardiography using a high‐frequency ultrasound system (Vevo 2100; VisualSonics Inc., Toronto, Ontario, Canada) with a 30‐MHz linear probe. 2D and M‐mode (MM) echography were performed at a frame rate above 230 frames per second, and pulsed wave Doppler was acquired with a pulse repetition frequency of 40 kHz. Mice were placed in a supine position on a heating platform, and warmed ultrasound gel was used to maintain normothermia. A base‐apex electrocardiogram was continuously monitored.

### Echocardiography analysis

Images were analyzed using Vevo 2100 Workstation software. For left ventricular (LV) systolic function assessment, parasternal standard 2D and MM, long‐ and short‐axis views (LAX and SAX view, respectively) were acquired. LV ejection fraction, LV fractional shortening, and LV chamber dimensions were calculated from these views.

## Results

### Identification of pathways affected by anesthesia

**Figure 1:**
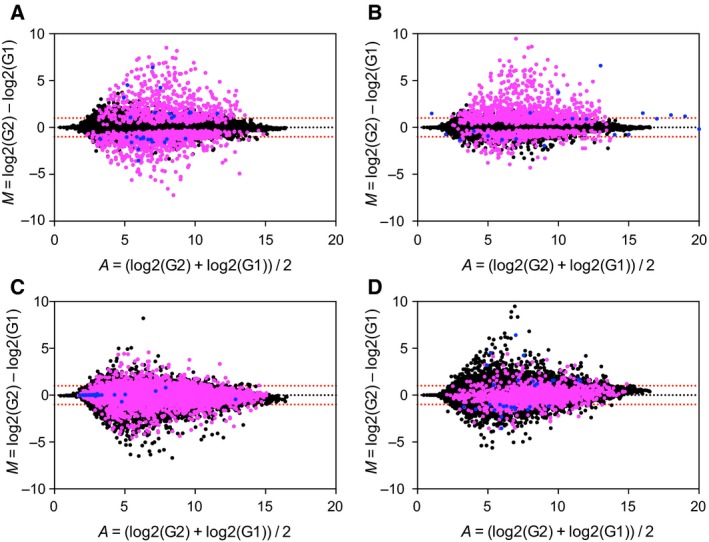
*In silico* analysis of gene expression changes in cardiomyocytes of patients administered Sevo and Prop. The MA plots show gene expression alterations. Pink dots represent genes with FDRs of < 0.05 in patients administered Sevo (A) and Prop (B) in unpaired test or Sevo (C) and Prop (D) in paired test. Blue dots represent genes downstream of NF‐kB identified in the analysis.

To study whether inhalational and/or intravenous anesthetics influence cardiac hypertrophy, we compared gene expression datasets in cardiac tissue following anesthesia with Prop and Sevo during OFF‐PUMP CABG surgery 16. The baseline characteristics of the patients are presented in Table 1. The cohort was divided into two groups according to which type of anesthetic was administered. The groups were comparable in terms of pre‐ and postoperative data such as comorbidities, medication, requirement of inotropic support, and complications including myocardial infarction and cerebrovascular injury 16. DEGs were identified as those with a FDR of < 0.05 and a LogFC of > 1 or < −1 using the unpaired test and the paired test to eliminate bias caused by the limited size of the cohort. The unpaired test identified 1002 and 390 DEGs in the Sevo‐ and Prop‐administered groups, respectively (Fig. 1A,B). The paired test identified 5249 and 2042 DEGs in the Sevo‐ and Prop‐administered groups, respectively (Fig. 1C,D).

**Table 1:** Baseline characteristics of patients in the Sevo‐ and Prop‐administered groups. This dataset was obtained from Lucchinetti *et al*. 16.

	Prop	Sevo
Age (years)		
Mean ± SD	66.9 ± 7.3	65.2 ± 7.6
Gender		
Male	10	10
Female	0	0

The identified DEGs were subjected to Ingenuity Pathway Analysis in order to define pathways that were up‐ or downregulated by anesthesia. Altered pathways were defined as those with a *P*‐value of < 0.05. The unpaired test and paired test revealed that 137 and 89 pathways, respectively, and 160 and 93 pathways, respectively, were dysregulated in response to Sevo and Prop (data not shown). The unpaired test and paired test revealed that only 14 pathways (10.2% and 15.7% in the Sevo‐ and Prop‐administered groups, respectively; Table 2) and nine pathways (5.6% and 9.7% in the Sevo‐ and Prop‐administered groups, respectively; Table 3), respectively, were differentially regulated by the two anesthetics. These data suggest that most pathways were altered in a similar manner following Sevo and Prop administration. Of the differentially dysregulated pathways (unpaired test, 14 pathways; paired test, nine pathways), NF‐kB signaling was the pathway whose activity differed the most between the two groups according to the results of the unpaired test and paired test (*z*‐score of −1.134 and +1.155 in the Sevo‐ and Prop‐administered groups, respectively; Tables 2 and 3). Bioinformatics analysis demonstrated that the NF‐kB signaling pathway was down‐ and upregulated by Sevo and Prop, respectively.

**Table 2:** Differentially altered pathways in patients administered Prop and Sevo identified using the unpaired test. *P*‐value was calculated using Fisher’s exact test.

Pathway	*P*‐value		Activation *z*‐score	
	Sevo	Prop	Sevo	Prop
NF‐κB	1.44E‐08	1.16E‐04	−1.134	1.155
CXCR4	8.78E‐03	3.09E‐02	−0.535	1.633
Gαq	9.07E‐05	2.14E‐03	−0.943	0.707
LPS‐stimulated MAPK	2.58E‐03	2.35E‐02	−0.302	1.342
Apoptosis	2.57E‐02	2.66E‐02	−1.000	0.447
Thrombopoietin	3.87E‐03	3.56E‐02	−0.333	1.000
Th2	1.54E‐02	4.93E‐03	−0.333	1.000
Prolactin	1.37E‐04	9.44E‐04	−0.832	0.378
STAT3	2.50E‐06	1.89E‐06	−0.243	0.905
mTOR	4.78E‐03	2.94E‐02	−0.535	0.447
Wnt/β‐catenin	2.02E‐02	3.17E‐02	−0.577	0.378
Activation of IRF by cytosolic pattern recognition receptors	2.05E‐02	4.85E‐03	−0.378	0.447
CD40	1.58E‐06	7.10E‐05	−0.277	0.378
ERK/MAPK	2.78E‐04	6.53E‐05	−0.218	0.277

**Table 3:** Differentially altered pathways in patients administered Prop and Sevo identified using the paired test. *P*‐value was calculated using Fisher’s exact test.

Pathway	*P*‐value		Activation *z*‐score	
	Sevo	Prop	Sevo	Prop
NF‐κB	1.00E‐08	5.86E‐05	−1.134	1.155
IL‐7	1.35E‐03	1.08E‐02	−1.265	1.000
Prolactin	3.33E‐05	2.65E‐03	−0.832	0.816
Apoptosis	2.06E‐02	2.63E‐02	−1.000	0.447
Gαq	2.34E‐05	3.64E‐04	−0.471	0.707
Th1	8.33E‐05	1.17E‐03	−0.535	0.447
Wnt/β‐catenin	3.98E‐02	4.21E‐03	−0.577	0.378
Activation of IRF by cytosolic pattern recognition receptors	3.10E‐02	4.21E‐03	−0.378	0.447
Endocannabinoid cancer inhibition	3.99E‐03	1.13E‐02	−0.258	−0.378

### The NF‐kB signaling pathway is altered in response to anesthesia

**Figure 2:**
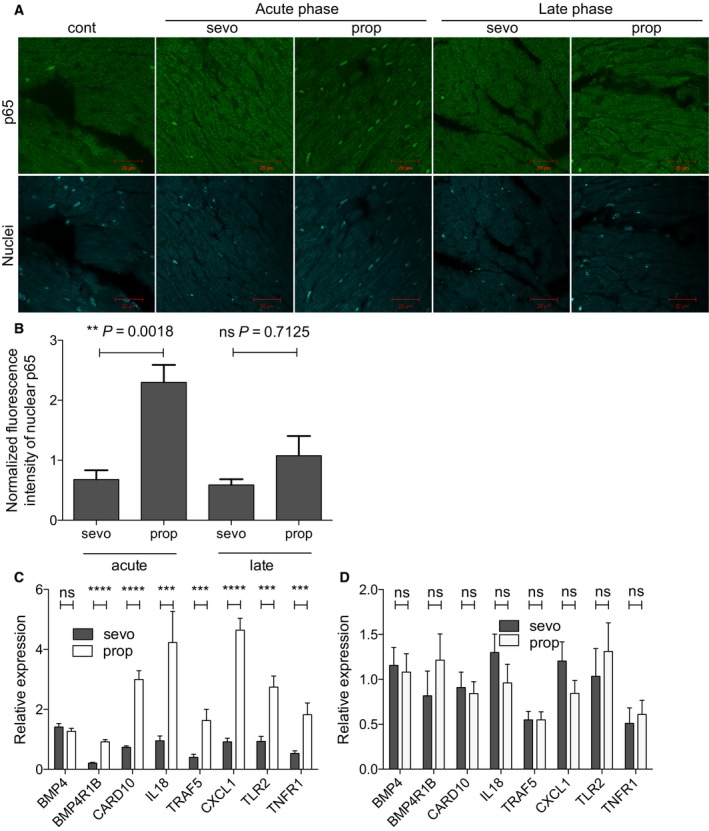
Effects of Sevo and Prop on NF‐kB signaling in cardiomyocytes of mice. (A) Representative images of p65 staining and nuclear staining in heart tissues of control, Sevo‐administered, and Prop‐administered mice in the acute phase (just after anesthesia) and the late phase (10 days after anesthesia). Scale bar indicates 20 μm. (B) Bar graph showing the average normalized mean fluorescence intensity of nuclear p65 staining in Sevo‐administered and Prop‐administered mice in the acute and the late phase. Error bars indicate standard errors. Four mice were investigated per group, four fields were studied per mouse, and 100 cells were examined per field. *P*‐value was calculated using the Mann–Whitney *U*‐test. (C, D) Bar graph showing mRNA expression of various genes downstream of NF‐kB in heart tissues of control, Sevo‐administered, and Prop‐administered mice in the acute phase (C) and the late phase (D). Error bars indicate standard errors. *P*‐value was calculated using the Mann–Whitney *U*‐test. ‘ns’, not significant; **P‐value < 0.005; ***P‐value < 0.0005; ****P‐value < 0.0001.

**Figure 3:**
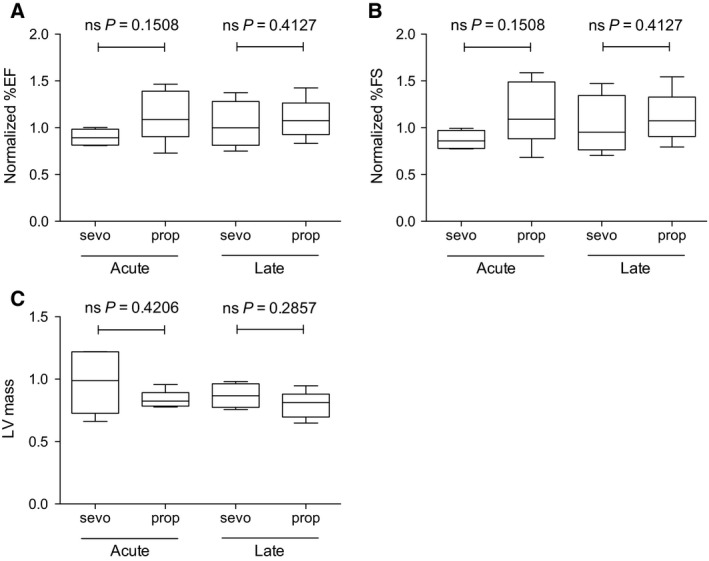
Effects of Sevo and Prop on cardiac function and left ventricle size in mouse cardiomyocytes. Box plot shows % ejection fraction (A) and % FS (B) and LV mass (C) of heart of control, Sevo‐administered, and Prop‐administered mice both in acute phase and in late phase. Error bars indicate minimum and maximum values. At least four mice were investigated per group.

Bioinformatics analysis of human samples indicated that activity of the NF‐kB signaling pathway was decreased by Sevo and increased by Prop in cardiac tissues (Fig. 1). To further investigate this, we examined the localization of p65, a core component of the NF‐kB complex that localizes to the nucleus when activated 22 in mice. Heart tissues were stained with an anti‐p65 antibody, and the nuclear localization of p65 was quantified using image analysis software. Nuclear localization of p65 was markedly decreased in the cardiomyocytes of mice in the early phase after administration of Sevo (Fig. 2A,B), but not in the late phase (Fig. 2A,B). Activated p65 localizes to the nucleus, where it binds to its response elements in target genes and promotes transcription to modulate cell proliferation, inflammation, and/or apoptosis 23. We confirmed the effect of anesthesia on the NF‐kB signaling pathway by measuring mRNA expression of downstream genes. Bioinformatics analysis revealed that mRNA expression of various genes downstream of NF‐kB was significantly lower in the Sevo‐administered group than in the Prop‐administered group (Table 4). Among the genes whose mRNA expression levels differed more than 1.5‐fold between the two groups, we analyzed mRNA expression of *BMP4*, *BMPR1B*, *CARD10*, *IL18*, and *TRAF5* in mice. We also examined mRNA expression of other target genes of NF‐kB, including *CXCL1*, *TLR2*, and *TNFR1*. This analysis confirmed that Sevo suppressed mRNA expression of genes downstream of NF‐kB (Fig. 2C). These data support our finding in bioinformatics analysis that the NF‐kB signaling pathway was down‐ and upregulated by Sevo and Prop, respectively. Interestingly, these effects were not observed in the late phase (Fig. 2D). We further studied whether changes in NF‐kB signaling induced by anesthesia affect cardiac function or left ventricle capacity measured by an echocardiogram. Although the two types of anesthesia differentially affected NF‐kB signaling, cardiac function and LV mass were not altered by either of these two types of anesthesia in the acute or late phase (Fig. 3A–C).

**Table 4:** Changes in mRNA expression of NF‐kB‐regulated genes in Sevo‐ and Prop‐anesthetized patients.

Gene	Paired LogFC		Unpaired LogFC	
	Sevo	Prop	Sevo	Prop
*BMP2*	1.568	1.484	1.568	1.484
*BMP4*	−1.561	0	−1.561	−0.744
*BMPR1B*	−1.597	0	−1.597	−1.346
*CARD10*	−1.592	0	−1.592	−0.385
*CASP8*	−1.289	0	−1.289	−0.913
*FADD*	−1.207	−1.316	−1.207	−1.316
*FGFR2*	−1.234	−1.226	−1.234	−1.226
*FLT1*	1.594	1.558	1.594	1.558
*IL18*	−3.535	0	−3.535	−1.947
*IL1B*	4.521	3.734	4.521	3.734
*IL1R1*	1.087	0	1.087	0.947
*IL1R2*	3.189	0	3.189	0.889
*IL1RN*	6.415	6.592	6.415	6.592
*IRS1*	−1.038	0	0	0
*KDR*	−1.196	0	−1.196	−0.758
*MAP3K8*	1.467	1.552	1.467	1.552
*NFKB1*	1.202	0	1.202	0.930
*NFKB1A*	1.487	1.333	1.487	1.333
*PELI1*	1.541	1.197	1.541	1.197
*PIK3C3*	−1.002	0	−1.002	−0.586
*PIK3C2A*	−1.026	0	−1.026	−0.183
*RIPK1*	1.055	1.154	1.055	1.154
*TLR3*	−1.287	0	−1.287	−0.841
*TLR5*	−1.155	0	−1.155	−0.856
*TLR7*	−1.244	0	−1.244	−0.446
*TNFAIP3*	4.244	4.288	4.244	4.288
*TNFRSF1B*	1.018	1.027	1.018	1.027
*TRAF5*	−2.331	0	−2.331	−0.876

## Discussion

Our unbiased bioinformatics analysis predicted that both inhalational and intravenous anesthetics affect myocardial remodeling through NF‐kB signaling because this signaling is inhibited by Sevo and promoted by Prop. We confirmed this finding in a mouse model. Although the mechanism by which Sevo and Prop affect cardiac function is unknown, our data provide a clue. Most pathways were similarly altered in the Sevo‐ and Prop‐administered groups; however, 10–15% of pathways were differentially regulated in these two groups (Tables 2 and 3). Among the differentially regulated pathways, activity of the NF‐kB signaling pathway differed the most between the two groups (Tables 2 and 3). Several lines of evidence suggest that NF‐kB plays a pivotal role in myocardial remodeling by regulating inflammation and cell death; however, additional pathways identified in our analysis, such as the Gαq and prolactin pathways (Tables 2 and 3), are also involved in such remodeling, especially in cardiac hypertrophy 24, 25. Our bioinformatics analysis and *in vivo* experiments (Figs 1 and 2), together with the previous finding that NF‐kB signaling elicits both cardioprotective and cardiotoxic effects 5, 6, suggest that NF‐kB signaling is one of the most important pathways underlying myocardial remodeling in response to anesthesia.

A previous study demonstrated that Sevo induces a lower inflammatory response than Prop during cardiac surgery 26. Also, a recent study revealed that Prop induces more inflammation than isoflurane, an inhalation anesthetic 27. These two studies investigated inflammatory responses by measuring release of cytokines, including IL‐6, p65, and TNF‐α, which are regulated by NF‐kB signaling. Our results are consistent with this previous report, and these findings suggest that Sevo suppresses NF‐kB signaling. Although representative genes downstream of NF‐kB, such as *CXCL1*, *TLR2*, and *TNFR1*, were not identified as DEGs by our bioinformatics analysis, experiments in mice demonstrated that mRNA expression of these genes was differentially regulated by Prop and Sevo. This finding supports our hypothesis that NF‐kB signaling plays an important role in the myocardial response to anesthesia. In our functional study, neither anesthetic affected cardiac function or ventricle capacity (Fig. 3). NF‐kB signaling plays an important role in cardiac remodeling, but takes considerable time, suggesting that anesthesia for 3 h may not be sufficient to observe the effect of anesthesia on cardiac function or capacity.

Activation of NF‐kB signaling is beneficial in the acute response to hypoxia and reperfusion injury, while prolonged activation is detrimental to the heart 5, 6. A recent report demonstrated that NF‐kB signaling plays a pivotal role in arrhythmogenic cardiomyopathy (ACM), which is an inherited disease that can cause sudden death 28. This report also showed that inhibition of NF‐kB signaling using BAY 11‐7082, a small molecule compound, suppresses ACM development. Thus, accumulated evidence suggests that NF‐kB is implicated in myocardial pathogenesis. However, it is unknown whether cardiomyopathy induced by anesthetics is important in the general population or confined only to patients with abnormal NF‐kB signaling at baseline, such as ACM patients. The observation that NF‐kB inhibition suppresses ACM development warrants an investigation into the effect of NF‐kB inhibition during anesthesia on cardiac complications, such as ACM. Our study provides a link between anesthesia and NF‐kB signaling changes in cardiomyocytes. These results could form the basis of a preclinical study to determine the optimal choice of anesthesia and the effect of NK‐kB inhibition together with Prop on cardia complications including ACM development. Because anesthetics, especially Prop, are used not only in surgery but also in intensive care, further investigations into the effects of anesthetics on myocardial function are urgently required.

## Conflict of interest

The authors declare no conflict of interest.

## Author contributions

ASS, JAB, and KS conceptualized the study, validated the data, and acquired funding; ASS and KS devised the methodology, carried out formal analysis, and wrote the original draft; ASS provided the software; KO‐K, ASS, NO, MI, KM, TI, TK, ES, EI, AY, MS, and MR‐M carried out investigation; ASS, KM, TK, JAB, and KS reviewed and edited the manuscript.
